# Overexpression of the Small RNA PA0805.1 in Pseudomonas aeruginosa Modulates the Expression of a Large Set of Genes and Proteins, Resulting in Altered Motility, Cytotoxicity, and Tobramycin Resistance

**DOI:** 10.1128/mSystems.00204-20

**Published:** 2020-05-19

**Authors:** Shannon R. Coleman, Maren L. Smith, Victor Spicer, Ying Lao, Neeloffer Mookherjee, Robert E. W. Hancock

**Affiliations:** a Center for Microbial Diseases and Immunity Research, Department of Microbiology and Immunology, University of British Columbia, Vancouver, Canada; b Manitoba Center for Proteomics & Systems Biology, University of Manitoba, Winnipeg, Canada; Leiden University

**Keywords:** *Pseudomonas aeruginosa*, antibiotic resistance, genomics, molecular genetics, motility, proteomics, swarming, virulence

## Abstract

Pseudomonas aeruginosa is a motile species that initiates swarming motility in response to specific environmental cues, i.e., a semisolid surface with amino acids as a nitrogen source (relevant to the human lung). Swarming is an intricately regulated process, but to date posttranscriptional regulation has not been extensively investigated. Small noncoding RNAs (sRNAs) are hypothesized to play posttranscriptional regulatory roles, largely through suppression of translation, and we previously demonstrated 20 sRNA species that were dysregulated under swarming conditions. One of these, sRNA PA0805.1 (which was 5-fold upregulated under swarming conditions), when cloned, transformed into wild-type (WT) PAO1, and overexpressed, led to broad phenotypic changes, including reduced swarming, swimming, and twitching motilities, as well as increased adherence, cytotoxicity, and tobramycin resistance. A ΔPA0805.1 deletion mutant was more susceptible to tobramycin than the WT under swarming conditions. The strain overexpressing PA0805.1 was compared to the empty-vector strain by transcriptome sequencing (RNA-Seq) and proteomics under swarming conditions to determine sRNA targets. Broad transcriptional and proteomic profiles showed 1,121 differentially expressed genes and 258 proteins with significantly different abundance. Importantly, these included 106 transcriptional regulators, two-component regulatory systems, and sigma and anti-sigma factors. Downstream of these regulators were found downregulated type IV pilus genes, many upregulated adherence and virulence factors, and two multidrug efflux systems, *mexXY* and *mexGHI-opmD*. Therefore, the sRNA PA0805.1 appears to be a global regulator that influences diverse bacterial lifestyles, most likely through a regulatory cascade.

**IMPORTANCE** P. aeruginosa is an opportunistic pathogen of humans. With roughly 10% of its genes encoding transcriptional regulators, and hundreds of small noncoding RNAs (sRNAs) interspersed throughout the genome, P. aeruginosa is able to fine-tune its response to adapt and survive in the host and resist antimicrobial agents. Understanding mechanisms of genetic regulation is therefore crucial to combat pathogenesis. The previously uncharacterized sRNA PA0805.1 was overexpressed in P. aeruginosa strain PAO1, resulting in decreased motility, increased adherence, cytotoxicity, and tobramycin resistance. In contrast, a ΔPA0805.1 deletion mutant had increased susceptibility to tobramycin under swarming conditions. Omic approaches uncovered 1,121 transcriptomic and 258 proteomic changes in the overexpression strain compared with the empty-vector strain, which included 106 regulatory factors. Downstream of these regulators were upregulated adherence factors, multidrug efflux systems, and virulence factors in both transcriptomics and proteomics. This study provides insights into the role of the sRNA PA0805.1 in modulating bacterial adaptations.

## Abstract (precis)

P. aeruginosa is an opportunistic pathogen of humans. With roughly 10% of its genes encoding transcriptional regulators, and hundreds of small noncoding RNAs (sRNAs) interspersed throughout the genome, P. aeruginosa is able to fine-tune its response to adapt and survive in the host and resist antimicrobial agents. Understanding mechanisms of genetic regulation is therefore crucial to combat pathogenesis. The previously uncharacterized sRNA PA0805.1 was overexpressed in P. aeruginosa strain PAO1, resulting in decreased motility, increased adherence, cytotoxicity, and tobramycin resistance. In contrast, a ΔPA0805.1 deletion mutant had increased susceptibility to tobramycin under swarming conditions. Omic approaches uncovered 1,121 transcriptomic and 258 proteomic changes in the overexpression strain compared with the empty-vector strain, which included 106 regulatory factors. Downstream of these regulators were upregulated adherence factors, multidrug efflux systems, and virulence factors in both transcriptomics and proteomics. This study provides insights into the role of the sRNA PA0805.1 in modulating bacterial adaptations.

## INTRODUCTION

Pseudomonas aeruginosa, a Gram-negative opportunistic and nosocomial pathogen, is highly motile and undergoes swarming motility in response to specific environmental conditions, namely, a semisolid environment with amino acids as a nitrogen source. These conditions are relevant to human lung diseases such as pneumonia and cystic fibrosis (CF). P. aeruginosa is a common CF pathogen, and the incidence of multidrug resistance has increased in recent years (1).

Swarming motility is a multicellular phenomenon, involving both pili and flagella, whereby groups of cells move in a concerted fashion by aligning with one another to propel themselves across a surface, occasionally branching out and resulting in rapid surface colonization and dendritic or solar-flare colonial structures. Swarming cells exhibit adaptive resistance to multiple antibiotic classes (2–4). Swarming is a highly regulated process, and previous studies have shown that 104 regulators are dysregulated under swarming conditions (4), while mutations in 35 regulators lead to alterations in swarming motility (5). However, alternative means of regulation have not been well investigated, including posttranscriptional and translational regulation and the modification or degradation of proteins.

Small RNAs (sRNAs) are noncoding RNA species, usually around 40 to 500 bp in length in P. aeruginosa, and are rapidly evolving (6). Typically they are thought to bind complementary mRNA to inhibit translation (7); however, sRNA-mRNA binding can also lead to translational activation or mRNA degradation (8). Translational repression is achieved by sRNAs blocking access to the ribosome binding site, whereas translational activation can result from sRNAs disrupting secondary structures in the mRNA in order to uncover ribosome binding sites (9). In addition, sRNAs can also bind to proteins and alter their activity (8, 10). sRNAs can be classified in two categories: *cis*- and *trans*-encoded. *Cis*-encoded sRNAs overlap their target mRNAs and have high sequence similarity, whereas *trans*-encoded sRNAs are distant from their targets and frequently utilize imperfect base pairing to mediate their effects (8). In many bacterial species, the RNA chaperone Hfq is required to stabilize sRNA-mRNA interactions (10), although *Pseudomonas* exhibits other more selective RNA-binding proteins, such as Crc and RsmA. Interestingly, prior to 2012 only 44 sRNAs had been identified in P. aeruginosa (6), but subsequent transcriptome sequencing (RNA-Seq) studies have identified hundreds of potential intergenic sRNAs (11, 12). Nevertheless, few of these novel sRNAs have been characterized, leaving a large field to be explored.

Prior research in our lab identified 20 sRNA species that were dysregulated under swarming conditions (13). One of these, PA0805.1, overlapping previously identified sRNAs pant89 (11) and PA14sr119/120 (12), was studied in this investigation in detail by genetic manipulation, phenotypic screens, and omic comparisons. PA0805.1 was chosen for further analysis based on phenotypic screens that showed an array of intriguing phenotypes, as well as expression data discussed in the next section.

## RESULTS

### Overexpression of PA0805.1 resulted in antimotility effects.

Specific quantitative reverse transcriptase PCR (qRT-PCR) analysis demonstrated that the transcript for PA0805.1 was upregulated under swarming conditions (versus swimming) 5.0-fold ± 1.7-fold. In contrast, it was downregulated in biofilm cells by 4.8-fold ± 3.8-fold (13). Since biofilms are considered a sedentary lifestyle typical of chronic infections while swarming is considered more typical of acute infections, this ∼25-fold difference in expression levels indicated that sRNA PA0805.1 had the potential to discriminate or even act as a switch between the chronic and acute modes of infection. To investigate this further, we overexpressed sRNA PA0805.1, since sRNAs often act in a suppressive manner (7). The PA0805.1 gene was cloned and inserted after the *araC* promoter in the arabinose-inducible pHERD20T vector and transformed into wild-type (WT) PAO1 H103. PA0805.1 was overexpressed after induction with arabinose 28.1-fold ± 1.9-fold under swarming conditions (BM2 glycerol, normalized to the housekeeping gene *rpoD*).

**FIG 1:**
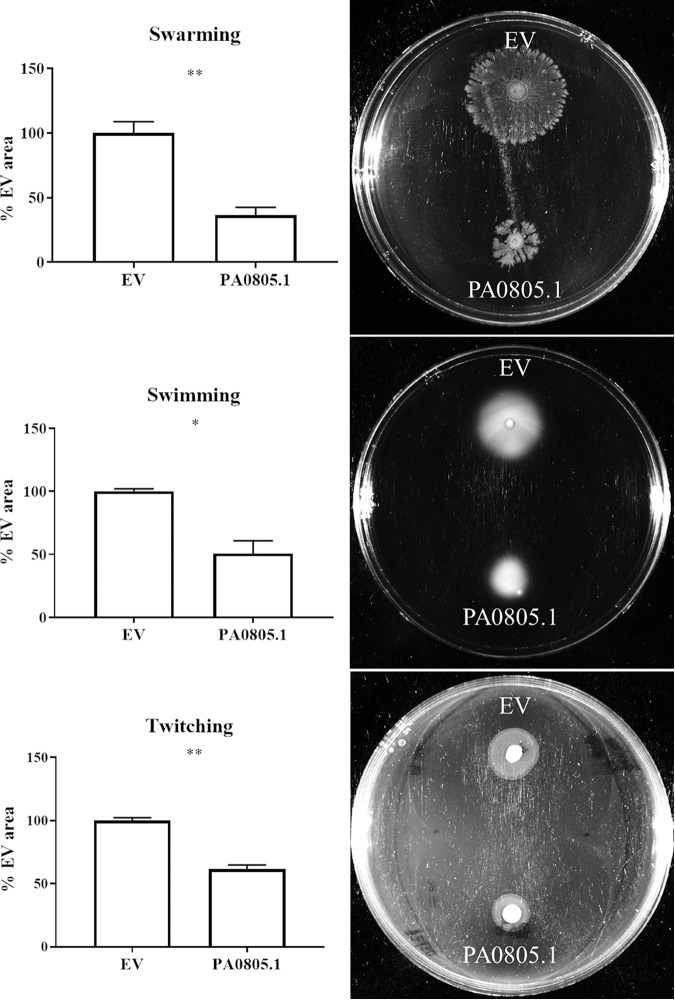
Motility assays revealed that overexpression of PA0805.1 was generally antimotility. Arabinose (1%) was used to induce expression, and statistically significant differences were determined using paired Student’s *t* test. *, 0.01 < *P* ≤ 0.05; **, 0.001 < *P* ≤ 0.01 (*n *≥* *3).

At the time of the assay, arabinose was added to induce expression. Motility assays showed that PA0805.1 overexpression had antimotility effects resulting in partial reductions in each of swarming (reduced to 36% ± 3% of EV), swimming (51% ± 10%), and twitching (61% ± 3%) motility (Fig. 1).

### Overexpression of PA0805.1 resulted in increased cytotoxicity against HBE cells and increased tobramycin (TOB) resistance.

**FIG 2:**
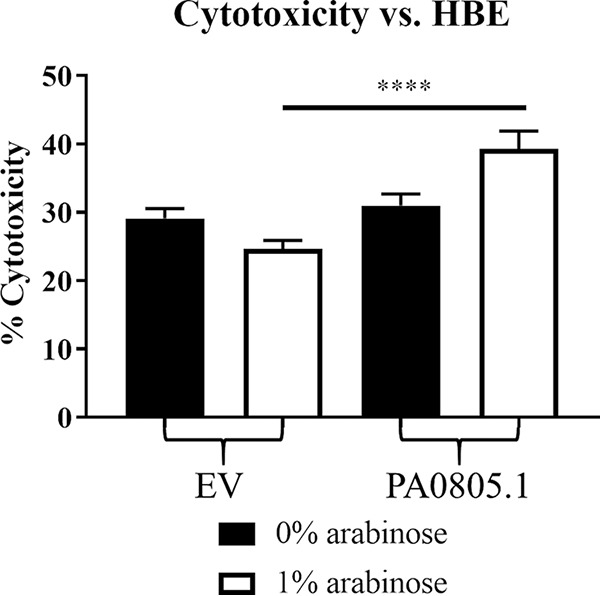
Cytotoxicity assay of the PA0805.1 overexpression strain revealing that induction of PA0805.1 led to increased cytotoxicity against HBE cells. Statistically significant differences were determined using paired Student’s *t* test. ****, *P* ≤ 0.0001 (*n *≥* *3).

The PA0805.1 overexpression strain was also tested for cytotoxicity against human bronchial epithelial 16HBE14o- (HBE) cells (Fig. 2). Overexpression of PA0805.1 resulted in a consistent and statistically significant phenotype, with a modest increase (15%) in cytotoxicity compared to that of the empty-vector (EV) strain. Growth curves performed in three different media (BM2, Dulbecco’s modified Eagle medium with l-glutamine and no d-glucose [DMEM] and LB) showed little difference between strains (see Fig. S1 in the supplemental material).

10.1128/mSystems.00204-20.1 FIG S1 Growth curves of PA0805.1 in three different media showed little difference from EV. *n *=* *3. Download FIG S1, TIF file, 0.2 MB. Copyright © 2020 Coleman et al. 2020 Coleman et al. This content is distributed under the terms of the Creative Commons Attribution 4.0 International license.

**FIG 3:**
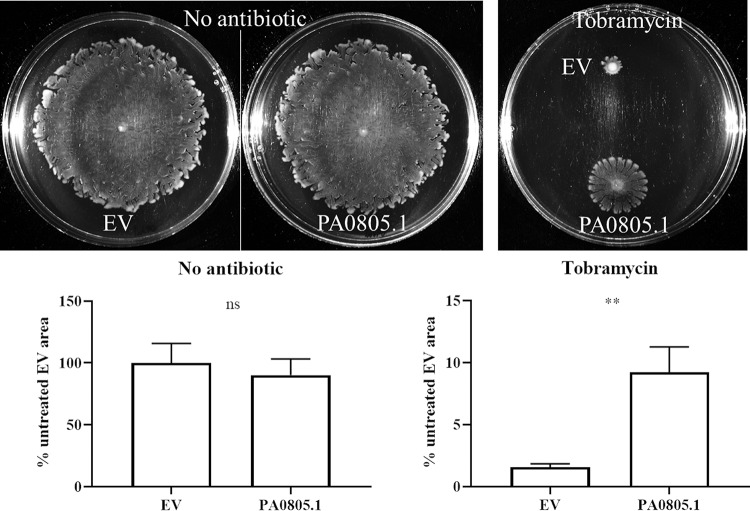
PA0805.1 overexpression led to swarming-dependent tobramycin resistance as assessed in BM2 0.4% glucose swarm plates with no arabinose and supplemented where indicated with tobramycin at 1 μg/ml. Statistically significant differences were determined using Student’s paired *t* test. **, 0.001 < *P* ≤ 0.01 (*n *=* *3). ns, not significant.

Following up from a previous study (4), we considered whether PA0805.1 might play a role in swarming-mediated antibiotic resistance. Therefore, the PA0805.1 overexpression strain was tested for tobramycin susceptibility under swarming conditions using a previously described method (4). Interestingly the PA0805.1 overexpression strain was resistant to tobramycin even in the absence of arabinose under swarming conditions (Fig. 3). In the presence of arabinose, the antimotility effect of PA0805.1 made it difficult to assess any antibiotic phenotypes under swarming conditions. To confirm that this phenotype was due to changes in antibiotic susceptibility, a tobramycin kill curve was performed, showing increased survival of swarm cells overexpressing PA0805.1 compared to that of EV swarm cells (Fig. S2). No consistent differences were observed between swimming cells overexpressing PA0805.1 and EV, indicating that the tobramycin phenotype may be specific to the swarming state (Fig. S2). We confirmed by qRT-PCR that PA0805.1 was overexpressed 6.2-fold ± 1.5-fold under swarming conditions (compared with the EV strain) in the absence of arabinose (BM2 glycerol, normalized to *rpoD*). MIC assays performed with the equivalent medium in microtiter trays showed little difference (Table S1).

10.1128/mSystems.00204-20.2 FIG S2 Tobramycin kill curve showed that swarm cells overexpressing PA0805.1 survived better than swarm cells with EV in the presence of tobramycin with no arabinose. Averages ± standard errors are depicted. Paired *t* tests were performed at each time point between EV swarm and PA0805.1 swarm to determine statistical differences. ***, 0.0001 < *P* ≤ 0.001; ****, *P* ≤ 0.0001 (*n *=* *3). Download FIG S2, TIF file, 0.5 MB. Copyright © 2020 Coleman et al. 2020 Coleman et al. This content is distributed under the terms of the Creative Commons Attribution 4.0 International license.

10.1128/mSystems.00204-20.4 TABLE S1 Tobramycin MIC (micrograms per milliliter) in BM2 glucose with 0.1% Casamino Acids and no (NH_4_)_2_SO_4_. (*n *=* *3). Download Table S1, DOCX file, 0.02 MB. Copyright © 2020 Coleman et al. 2020 Coleman et al. This content is distributed under the terms of the Creative Commons Attribution 4.0 International license.

### Overexpression of PA0805.1 resulted in broad protein and transcriptional changes, including 106 regulatory factors.

**FIG 4:**
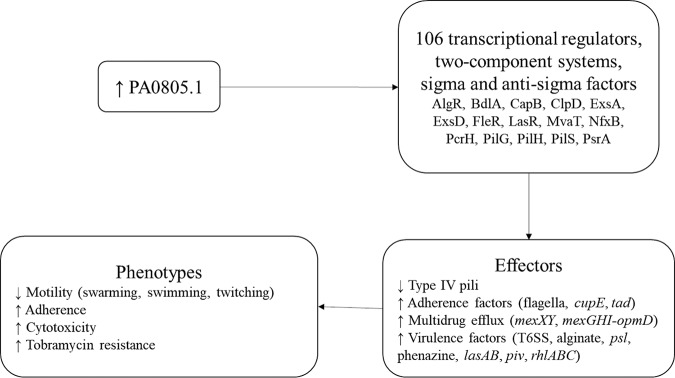
Proposed model for how the overexpression of PA0805.1 dysregulated many genes, resulting in altered phenotypes. Connecting arrows represent direct or indirect regulation.

To investigate these phenotypes further, we performed proteomics and RNA-Seq under swarming conditions in the presence of arabinose compared to EV as a control. Proteomics identified 258 proteins with significantly different abundance (*P* ≤ 0.05 and absolute fold change [FC] ≥ 1.25) in the PA0805.1 overexpression strain compared to EV, including 140 with increased abundance and 118 with decreased abundance (Table S2). In addition, there were 1,121 differentially expressed (DE) genes (*P* ≤ 0.05 and absolute FC ≥ 1.5) revealed by RNA-Seq, with 401 downregulated and 720 upregulated (Table S2). Among the DE genes and proteins with differential abundance, 106 transcriptional regulators, two-component systems, and sigma and anti-sigma factors were found (Table 1). These changes thus might explain in part the rather substantial transcriptional dysregulation observed. Some of the most strongly dysregulated regulators at the protein level (that were also transcriptionally dysregulated) included CapB, ClpD, ExsD, and PilGH. Many other important regulators involved in diverse processes, such as virulence, antimicrobial resistance, and quorum sensing, were also represented on the list of dysregulated regulators, including CprS, LasR, MvaT, PsrA, RhlR, and RsaL. Other dysregulated regulators that were upstream of the widespread changes in motility, adherence, and virulence factors described below included FleR, PilS, and AlgR (Fig. 4).

**TABLE 1:** Selected genes of interest with differential expression in the PA0805.1 overexpression strain compared to EV by RNA-Seq and/or proteomics^*a*^

Category	Locus tag	Name	Product name	RNA-Seq		Proteomics	
				FC	*P*_adj_	FC	*P*
Transcriptional regulators, two-component systems, sigma and anti-sigma factors	PA0150		Anti-sigma factor	−1.71	2.9E−02		
	PA0178		Probable two-component sensor	1.83	8.5E−06		
	PA0179		Probable two-component response regulator	2.02	2.0E−06		
	PA0268		Probable transcriptional regulator	1.52	3.0E−03		
	PA0408	*pilG*	Twitching motility protein	−2.09	2.8E−17	−1.52	2.5E−03
	PA0409	*pilH*	Twitching motility protein	−2.42	2.2E−29	−1.64	1.2E−02
	PA0416	*chpD*	Probable transcriptional regulator	−1.96	9.0E−07		
	PA0459	*clpD*	Probable Clpa/B protease ATP binding subunit	1.70	3.3E−04	1.89	9.0E−04
	PA0471	*fiuR*	Sigma factor	−1.80	4.6E−04		
	PA0472	*fiuI*	Sigma factor related	−2.03	4.2E−04		
	PA0479		Probable transcriptional regulator	1.57	2.5E−04		
	PA0535		Probable transcriptional regulator	−1.69	1.2E−03		
	PA0612	*ptrB*	Repressor	2.52	6.3E−04		
	PA0757		Probable two-component sensor	−1.53	9.9E−04		
	PA0763	*mucA*	Anti-sigma factor	1.68	5.7E−05		
	PA0807	*ampDh3*	Regulator of β-lactamase activity	2.18	8.3E−07		
	PA0929		Two-component response regulator	−1.68	1.9E−03		
	PA0930		Two-component sensor	−1.61	1.5E−02		
	PA0942		Probable transcriptional regulator	1.54	1.2E−03		
	PA0964	*pmpR*	*pqsR*-mediated PQS regulator	−1.64	8.6E−08	−1.18	3.2E−04
	PA1099	*fleR*	Two-component response regulator	1.52	3.9E−08		
	PA1136		Probable transcriptional regulator	1.75	1.4E−04		
	PA1159		Probable cold shock protein (DNA-binding domain)			−1.44	2.4E−02
	PA1363		ECF sigma factor	−1.69	2.4E−03		
	PA1364		Probable transmembrane sensor	−1.88	1.3E−02		
	PA1397		Probable two-component response regulator	−1.68	5.3E−05		
	PA1405		Probable helicase	−1.69	4.9E−04		
	PA1423	*bdlA*	Chemotaxis transducer	1.94	1.4E−06		
	PA1430	*lasR*	Transcriptional regulator			−1.28	4.9E−02
	PA1431	*rsaL*	Regulatory protein	1.70	6.8E−04	1.25	8.3E−03
	PA1619		Probable transcriptional regulator	−1.50	1.8E−03		
	PA1705	*pcrG*	Regulator in type III secretion	−9.28	5.4E−12		
	PA1707	*pcrH*	Regulatory protein	−8.60	4.9E−26		
	PA1713	*exsA*	Transcriptional regulator	−4.77	6.3E−62		
	PA1714	*exsD*	Negative regulator	−3.75	8.1E−34	−1.87	8.1E−04
	PA1785	*nasT*	Regulatory protein	1.76	3.3E−02	−1.26	1.3E−04
	PA1859		Probable transcriptional regulator	1.55	5.8E−04		
	PA1945		Probable transcriptional regulator	1.53	3.6E−03		
	PA2126	*cgrC*	CupA gene regulator C	2.18	1.2E−05		
	PA2126.1	*cgrB*	CupA gene regulator B	1.78	7.6E−03		
	PA2127	*cgrA*	CupA gene regulator A	1.80	4.3E−06		
	PA2177		Probable sensor/response regulator hybrid	1.77	2.5E−04		
	PA2227	*vqsM*	AraC-type transcriptional regulator	1.77	3.3E−06		
	PA2258	*ptxR*	Transcriptional regulator	2.15	1.2E−05		
	PA2259	*ptxS*	Transcriptional regulator	1.82	1.6E−04		
	PA2273	*soxR*	Regulatory protein	2.38	1.5E−09		
	PA2276		Probable transcriptional regulator	3.41	2.8E−38	1.27	9.3E−04
	PA2277	*arsR*	Regulatory protein	2.28	1.9E−06		
	PA2376		Probable transcriptional regulator	1.56	1.4E−03		
	PA2388	*fpvR*	Regulatory protein			−1.30	1.2E−02
	PA2467	*foxR*	Anti-sigma factor	−1.62	1.3E−02		
	PA2523	*czcR*	Regulatory protein	2.32	4.9E−08		
	PA2524	*czcS*	Regulatory protein	2.24	1.2E−04		
	PA2571		Probable two-component sensor	2.02	3.1E−06		
	PA2572		Probable two-component response regulator	1.86	8.1E−06	1.29	1.4E−02
	PA2577		Probable transcriptional regulator	1.70	3.4E−05		
	PA2620	*clpA*	ATP-binding protease component	1.60	4.8E−06	1.16	1.1E−02
	PA2622	*cspD*	Cold shock protein	1.80	1.5E−05	1.25	6.0E−03
	PA2696		Probable transcriptional regulator	1.53	4.5E−03		
	PA2771		Diguanylate cyclase	1.84	3.8E−09		
	PA2846		Probable transcriptional regulator	1.64	7.3E−03		
	PA2849	*ohrR*	Regulatory protein	1.63	2.2E−05	1.11	2.8E−02
	PA2889	*atvR*	Atypical virulence-related response regulator	1.55	7.7E−04		
	PA3006	*psrA*	Transcriptional regulator			1.09	3.4E−02
	PA3007	*lexA*	Repressor protein	1.84	5.6E−23	1.10	6.1E−03
	PA3078	*cprS*	Sensor kinase	−1.50	1.3E−03		
	PA3160	*wzz*	O-antigen chain length regulator			1.27	2.3E−02
	PA3174	*hutR*	Regulatory protein	−1.66	1.8E−02		
	PA3266	*capB*	Cold acclimation protein B	−1.54	1.1E−04	−1.67	3.2E−02
	PA3346	*hsbR*	HptB-dependent secretion and biofilm regulator	1.78	3.6E−09	1.22	1.2E−02
	PA3347	*hsbA*	HptB-dependent secretion and biofilm anti-anti-sigma factor	1.66	4.9E−05		
	PA3477	*rhlR*	Transcriptional regulator	1.77	1.9E−05	1.17	1.9E−02
	PA3622	*rpoS*	Sigma factor	1.75	1.4E−09	1.17	5.1E−03
	PA3899	*fecI*	Regulatory protein	−1.73	1.3E−02		
	PA3946	*rocS1*	Two-component sensor	1.74	4.3E−07		
	PA3947	*rocR*	Regulatory protein	1.57	7.5E−06		
	PA4070		Probable transcriptional regulator	1.52	3.3E−02		
	PA4074		Probable transcriptional regulator	2.20	2.7E−03		
	PA4080		Probable response regulator	1.54	5.3E−04		
	PA4203	*nmoR*	Regulatory protein	1.50	4.6E−02		
	PA4218	*ampP*	Regulator of β-lactamase activity	−1.77	1.5E−05	−1.18	1.8E−02
	PA4219	*ampO*	Regulator of β-lactamase activity	−1.76	4.3E−04		
	PA4288		Probable transcriptional regulator	2.20	1.9E−07		
	PA4293	*pprA*	Two-component sensor	2.22	5.0E−16	1.31	3.8E−03
	PA4296	*pprB*	Two-component response regulator	1.82	6.4E−07	1.12	1.1E−03
	PA4315	*mvaT*	Transcriptional regulator mvat, P16 subunit			1.26	2.6E−02
	PA4396		Two-component response regulator	−2.15	4.8E−11		
	PA4464	*ptsN*	Nitrogen-regulatory IIA protein	1.74	1.6E−10		
	PA4493	*roxR*	Regulatory protein			1.26	2.5E−02
	PA4546	*pilS*	Two-component sensor			1.28	3.1E−03
	PA4600	*nfxB*	Transcriptional regulator			1.25	4.7E−02
	PA4601	*morA*	Motility regulator	−1.54	1.2E−12		
	PA4624	*cdrB*	Cyclic diguanylate-regulated TPS partner B	2.27	1.4E−31	1.25	1.7E−02
	PA4625	*cdrA*	Cyclic diguanylate-regulated TPS partner A	4.18	7.6E−58		
	PA4769		Probable transcriptional regulator			1.35	2.8E−02
	PA4781		Cyclic di-GMP phosphodiesterase	1.58	2.0E−04		
	PA4843	*gcbA*	Regulatory protein	−1.97	4.4E−18		
	PA4886		Probable two-component sensor	−1.65	2.3E−02		
	PA4916	*nrtR*	Nudix-related transcriptional regulator	1.60	1.8E−08	1.18	4.6E−03
	PA4959	*fimX*	Response regulator and diguanylate cyclase	−1.54	2.7E−07		
	PA4969	*cpdA*	Cyclic AMP phosphodiesterase	−1.80	1.6E−08		
	PA5117	*typA*	Regulatory protein	−1.56	2.3E−05		
	PA5261	*algR*	Alginate biosynthesis regulatory protein	1.57	1.4E−05		
	PA5274	*rnk*	Nucleoside diphosphate kinase regulator	−1.53	1.5E−05	−1.26	3.5E−02
	PA5356	*glcC*	Transcriptional regulator	1.74	7.2E−07		
	PA5380	*gbdR*	Regulatory protein	1.58	2.5E−02		
	
Multidrug efflux systems	PA2018	*mexY*	Resistance-nodulation-cell division (RND) multidrug efflux transporter	1.79	6.5E−04		
	PA2019	*mexX*	RND multidrug efflux membrane fusion protein	1.91	4.9E−07	1.53	5.4E−04
	PA4205	*mexG*	Hypothetical protein	1.76	2.0E−14	1.59	9.1E−05
	PA4206	*mexH*	Probable RND efflux membrane fusion protein	1.71	7.2E−13	1.11	2.8E−02
	
Motility and related genes	PA0020	*tsaP*	T4P secretin-associated protein	−1.97	6.8E−11	−1.35	1.0E−03
	PA0410	*pilI*	Twitching motility protein	−2.37	1.1E−16	−1.48	6.8E−04
	PA0411	*pilJ*	Twitching motility protein	−2.76	4.3E−24	−1.49	4.8E−05
	PA0412	*pilK*	Methyltransferase	−2.40	2.3E−19		
	PA0413	*chpA*	Pilus-related chemotactic signal transduction system component	−2.44	1.9E−33	−1.24	3.8E−06
	PA0414	*chpB*	Probable methylesterase	−2.31	3.1E−24		
	PA0415	*chpC*	Probable chemotaxis protein	−2.18	2.0E−13		
	PA0417	*chpE*	Probable chemotaxis protein	−2.77	7.8E−04		
	PA0499		Probable pilus assembly chaperone	1.87	3.1E−03		
	PA1077	*flgB*	Flagellar basal-body rod protein	1.66	5.3E−11		
	PA1078	*flgC*	Flagellar basal-body rod protein	1.70	5.1E−10		
	PA1079	*flgD*	Flagellar basal-body rod modification protein	1.56	8.1E−09		
	PA1080	*flgE*	Flagellar hook protein	1.58	9.5E−11		
	PA1081	*flgF*	Flagellar basal-body rod protein	1.65	5.1E−11		
	PA1082	*flgG*	Flagellar basal-body rod protein	1.57	4.5E−07		
	PA1084	*flgI*	Flagellar P-ring protein precursor	1.53	1.6E−08		
	PA1085	*flgJ*	Flagellar protein	1.54	2.4E−06		
	PA1092	*fliC*	Flagellin type B	1.55	4.7E−06		
	PA1094	*fliD*	Flagellar capping protein	1.53	4.9E−06		
	PA1100	*fliE*	Flagellar hook-basal body complex protein	1.76	6.3E−08		
	PA1101	*fliF*	Flagellar M-ring outer membrane protein precursor	1.52	3.5E−11		
	PA1130	*rhlC*	Rhamnosyltransferase 2	1.56	5.8E−04		
	PA1452	*flhA*	Flagellar biosynthesis protein			1.28	1.3E−02
	PA3350		Hypothetical protein	1.62	3.1E−15	1.17	4.2E−03
	PA3351	*flgM*	Flagellar anti-sigma factor	1.52	1.2E−07		
	PA3478	*rhlB*	Rhamnosyltransferase chain B	2.39	3.0E−04		
	PA3479	*rhlA*	Rhamnosyltransferase chain A	2.55	1.1E−04		
	PA3526	*motY*	Flagellar motor protein	1.60	2.3E−07		
	PA4085	*cupB2*	Chaperone	1.52	3.7E−02		
	PA4294		Hypothetical protein	2.32	1.4E−20		
	PA4295	*fppA*	Flp prepilin peptidase A	1.68	9.8E−05		
	PA4297	*tadG*	Putative Tad-like Flp pilus-assembly protein	1.53	1.1E−02		
	PA4298		Hypothetical protein	2.25	1.9E−07		
	PA4299	*tadD*	Flp pilus assembly lipoprotein	1.89	1.4E−06	1.23	3.0E−03
	PA4300	*tadC*	Flp pilus assembly protein TadC	1.78	1.2E−05	1.29	8.1E−03
	PA4301	*tadB*	Flp pilus assembly protein	1.95	1.5E−06		
	PA4302	*tadA*	ATPase	1.93	2.8E−08	1.20	5.0E−02
	PA4303	*tadZ*	Pilus assembly protein	2.09	5.3E−11	1.22	1.4E−02
	PA4304	*rcpA*	Secretin	1.97	1.3E−10		
	PA4305	*rcpC*	Flp pilus assembly protein	2.12	8.4E−10		
	PA4306	*flp*	Type IVb pilin	1.84	1.4E−03		
	PA4525	*pilA*	Type 4 fimbrial precursor	−1.64	2.0E−04	−1.30	3.4E−03
	PA4528	*pilD*	Type 4 prepilin peptidase	−1.84	1.1E−08		
	PA4550	*fimU*	Type 4 fimbrial biogenesis protein	−1.74	2.0E−06	−1.11	4.9E−02
	PA4551	*pilV*	Type 4 fimbrial biogenesis protein	−1.99	9.3E−07	−1.10	1.7E−02
	PA4552	*pilW*	Type 4 fimbrial biogenesis protein	−1.72	9.2E−08	−1.22	2.4E−03
	PA4553	*pilX*	Type 4 fimbrial biogenesis protein	−1.63	1.4E−04		
	PA4554	*pilY1*	Type 4 fimbrial biogenesis protein	−1.59	9.6E−08		
	PA4555	*pilY2*	Type 4 fimbrial biogenesis protein	−1.51	6.9E−05	−1.34	7.3E−03
	PA4556	*pilE*	Type 4 fimbrial biogenesis protein			−1.64	9.4E−03
	PA4648	*cupE1*	Pilin subunit	2.56	2.7E−16		
	PA4649	*cupE2*	Pilin subunit	2.06	1.8E−12		
	PA4650	*cupE3*	Pilin subunit	1.91	1.9E−06		
	PA4651	*cupE4*	Pilin assembly chaperone	2.00	5.2E−20	1.28	1.3E−03
	PA4652	*cupE5*	Fimbrial usher protein	1.66	1.7E−07		
	PA4653	*cupE6*	Adhesin-like protein	1.69	1.1E−06		
	PA4959	*fimX*	Diguanylate cyclase/phosphodiesterase	−1.54	2.7E−07		
	PA5040	*pilQ*	Type 4 fimbrial biogenesis outer membrane protein	−1.63	8.0E−11	−1.38	4.7E−03
	PA5041	*pilP*	Type 4 fimbrial biogenesis protein	−1.70	1.2E−08		
	PA5042	*pilO*	Type 4 fimbrial biogenesis protein	−1.67	2.6E−12	−1.26	9.0E−04
	PA5043	*pilN*	Type 4 fimbrial biogenesis protein	−1.70	2.1E−10	1.77	6.6E−04
	PA5044	*pilM*	Type 4 fimbrial biogenesis protein	−1.53	1.5E−10	−1.27	5.6E−03
	
Type VI secretion system	PA0071	*tagR1*	FGE-sulfatase domain-containing protein			1.37	1.8E−03
	PA0075	*pppA*	Serine/threonine protein phosphatase			1.45	1.1E−02
	PA0076	*tagF1*	Type VI secretion-associated protein	1.56	1.5E−02		
	PA0077	*icmF1*	Type VI secretion protein			1.37	4.7E−04
	PA0078	*tssL1*	Type VI secretion system protein	1.59	1.3E−03	1.31	5.6E−03
	PA0079	*tssK1*	Type VI secretion protein	1.58	1.4E−04	1.40	3.1E−03
	PA0080	*tssJ1*	Type VI secretion protein	1.51	1.7E−07		
	PA0082	*tssA1*	Type VI secretion protein	1.52	4.1E−05	1.48	2.7E−03
	PA0083	*tssB1*	Type VI secretion protein	1.72	2.8E−06		
	PA0084	*tssC1*	Type VI secretion protein	1.58	5.7E−04	1.52	4.7E−03
	PA0085	*hcp1*	Type VI secretion system effector	1.74	3.9E−05	2.14	4.5E−04
	PA0086	*tagJ1*	Type VI secretion system	1.74	1.6E−04		
	PA0087	*tssE1*	Type VI secretion system lysozyme-like protein	1.92	1.7E−04		
	PA0088	*tssF1*	Type VI secretion protein	1.58	3.7E−03		
	PA0090	*clpV1*	Chaperone	1.59	1.5E−03	1.54	3.4E−03
	PA0091	*vgrG1*	Type VI secretion system tip protein	1.52	6.0E−03	1.23	2.4E−04
	PA0094	*eagT6*	Chaperone			1.38	4.7E−04
	PA0095		Type VI secretion protein	1.57	4.4E−09		
	PA0096		Hypothetical protein	2.11	1.4E−06		
	PA0097		Hypothetical protein	1.63	2.0E−07		
	PA0098		Hypothetical protein	1.67	6.2E−04		
	PA0099		Type vi effector protein	1.57	6.4E−06		
	PA0100		Hypothetical protein	1.52	2.0E−06	1.29	4.4E−04
	PA1659	*hsiF2*	Type VI secretion system lysozyme-like protein	1.61	1.3E−04		
	PA1661	*hsiH2*	Type VI secretion protein	1.53	8.1E−03		
	PA1666	*lip2*	Type VI secretion system lipoprotein			1.33	1.8E−04
	PA2361	*icmF3*	Type VI secretion protein	1.52	1.6E−05		
	PA2362	*dotU3*	Type VI secretion protein	1.89	1.1E−05		
	PA2363	*hsiJ3*	Type VI secretion protein	1.71	1.8E−09		
	PA2364	*lip3*	Type VI secretion protein	1.55	7.4E−05		
	PA2365	*hsiB3*	Type VI secretion protein	1.86	7.4E−09		
	PA2366	*hsiC3*	Type VI secretion protein	1.88	1.8E−07		
	PA2367	*hcp3*	Type VI secretion system effector	1.76	2.2E−06		
	PA2368	*hsiF3*	Type VI secretion protein	1.68	3.1E−03		
	PA2369	*hsiG3*	Type VI secretion protein	1.92	3.2E−11		
	PA2370	*hsiH3*	Type VI secretion protein	2.20	1.9E−06		
	PA2371	*clpV3*	Type VI secretion system ATPase	1.65	9.5E−07		
	PA2372		Hypothetical protein	1.69	1.4E−05		
	PA2373	*vgrG3*	Type VI secretion protein	1.68	4.7E−07		
	PA3486	*vgrG4b*	Type VI secretion protein	1.69	6.9E−04		
	PA5266	*vgrG6*	Type VI secretion protein	1.89	7.3E−04		
	
Other virulence factors	PA0051	*phzH*	Potential phenazine-modifying enzyme	2.53	9.4E−13		
	PA0122	*rahU*	Hemolysin	2.30	3.0E−07	2.04	1.4E−02
	PA1871	*lasA*	LasA protease precursor	1.68	2.0E−04		
	PA1899	*phzA2*	Probable phenazine biosynthesis protein	1.70	2.6E−08		
	PA1900	*phzB2*	Probable phenazine biosynthesis protein	1.95	3.0E−10	1.25	4.3E−02
	PA1901	*phzC2*	Phenazine biosynthesis protein	1.85	2.2E−06	1.44	4.5E−03
	PA1903	*phzE2*	Phenazine biosynthesis protein	1.24	7.2E−04		
	PA1905	*phzG2*	Probable pyridoxamine 5′-phosphate oxidase	1.53	2.7E−05	1.16	9.7E−03
	PA2231	*pslA*	Undecaprenyl-phosphate glucose phosphotransferase	1.85	1.3E−09	1.29	2.2E−03
	PA2232	*pslB*	Mannose-1-phosphate guanylyltransferase/mannose-6-phosphate isomerase	1.80	8.7E−12	1.34	6.5E−03
	PA2233	*pslC*	Putative glycosyl transferase	1.58	5.4E−09	1.14	4.5E−02
	PA2234	*pslD*	Polysaccharide export protein	1.71	4.9E−18	1.29	5.6E−03
	PA2235	*pslE*	Psl exopolysaccharide biosynthesis	1.68	2.6E−09	1.19	4.8E−03
	PA2236	*pslF*	Glycosyl transferase	1.62	1.4E−07		
	PA2237	*pslG*	Beta-xylosidase	1.52	1.4E−14	1.19	4.1E−03
	PA2238	*pslH*	Glycosyl transferase	1.71	1.1E−06	1.28	1.8E−03
	PA2239	*pslI*	Psl exopolysaccharide biosynthesis	1.55	7.4E−05	1.13	4.6E−02
	PA2243	*pslM*	Hypothetical protein	1.73	5.1E−03		
	PA2244	*pslN*	Hypothetical protein	1.88	8.2E−03		
	PA2570	*lecA*	Galactose-binding lectin	7.32	2.4E−08	2.41	3.4E−03
	PA3361	*lecB*	Fucose-binding lectin PA-IIL	2.86	1,2E−07		
	PA3540	*algD*	GDP-mannose 6-dehydrogenase	7.13	2.9E−09		
	PA3541	*alg8*	Alginate biosynthesis protein	2.79	4.7E−05		
	PA3542	*alg44*	Alginate biosynthesis protein	2.11	2.7E−02		
	PA3544	*algE*	Alginate production outer membrane protein	2.73	1.8E−04		
	PA3545	*algG*	Alginate-c5-mannuronan-epimerase	2.41	9.7E−05		
	PA3547	*algL*	Poly(beta-d-mannuronate) lyase precursor	2.03	3.5E−02		
	PA3548	*algI*	Alginate *o*-acetyltransferase	1.80	3.9E−02		
	PA3550	*algF*	Alginate *o*-acetyltransferase	1.74	4.7E−02		
	PA3551	*algA*	Phosphomannose isomerase/GDP-d-mannose pyrophosphorylase	1.82	1.3E−03		
	PA3724	*lasB*	Elastase	1.64	1.7E−07		
	PA4175	*piv*	Protease IV	1.86	1.7E−05		
	PA4212	*phzC1*	Phenazine biosynthesis protein			1.44	4.5E−03
	PA4213	*phzD1*	Phenazine biosynthesis protein	1.20	1.4E−03		
	PA4214	*phzE1*	Phenazine biosynthesis protein	1.24	7.2E−04		

a Categories of interest include regulators, multidrug efflux, motility, type VI secretion system,and other virulence factors. Cutoffs used were *P* value of ≤0.05, absolute FC of ≥1.5 for RNA-Seq, and absolute FC of ≥1.25 for proteomics, although proteins with FC of ≤1.25 are also shown if there was a corresponding RNA-Seq or qRT-PCR value. *n *≥* *3. *P*_adj_, adjusted *P* value.

10.1128/mSystems.00204-20.5 TABLE S2 Transcripts and proteins with differential abundance in PA0805.1 versus EV by RNA-Seq and proteomics. Two analyses are shown for reference using proteomic cutoffs of (i) *P* ≤ 0.05 and (ii) *P* ≤ 0.05 and absolute FC ≥ 1.25. Download Table S2, XLSX file, 0.6 MB. Copyright © 2020 Coleman et al. 2020 Coleman et al. This content is distributed under the terms of the Creative Commons Attribution 4.0 International license.

### The multidrug efflux genes *mexXY* and *mexGHI-opmD* were upregulated in the PA0805.1 overexpression strain.

Related to the tobramycin phenotype, the multidrug efflux pump *mexXY*, a known efflux pump mediating resistance to aminoglycosides (14), was upregulated (Table 1). We verified by qRT-PCR that *mexX* was upregulated 3.1-fold (Table S3). In addition, the multidrug efflux genes *mexGH* were upregulated in the RNA-Seq and proteomic data, which could contribute to tobramycin resistance, since aminoglycosides are a substrate of the MexGHI-OpmD pump (Table 1) (15). qRT-PCR indeed showed that the whole *mexGHI-opmD* operon was mildly upregulated (Tables 1 and 2 and Table S2). Furthermore, *czcR*, a response regulator involved in heavy metal resistance, was upregulated 2.3-fold in the RNA-Seq (Table 1). Moreover, *wbpX* and *wbpY* (involved in lipopolysaccharide [LPS] biosynthesis) were downregulated, and in other studies we showed that these can also lead to tobramycin resistance (Table S2) (4, 16). In contrast, genes in a different LPS biosynthetic operon (*wbpM*, *wbpE*, and *wzz*) were upregulated (Table S2). To confirm RNA-Seq and proteomics results, the expression of seven more genes was verified by qRT-PCR (Table S3).

**TABLE 2:** The MexGHI-OpmD operon was upregulated in the PA0805.1 overexpression strain compared to the EV strain^*a*^

Gene	Fold change
*mexG*	2.1 ± 0.1
*mexH*	2.0 ± 0.2
*mexI*	1.9 ± 0.1
*opmD*	2.1 ± 0.4

a Bacteria were harvested from BM2 swarm plates with 0.4% glycerol, 1% arabinose, and 0.1% Casamino Acids, and qRT-PCR was performed. Means ± standard errors are shown (*n *=* *3), normalized to the housekeeping gene *rpoD*.

10.1128/mSystems.00204-20.6 TABLE S3 Additional genes verified by qRT-PCR. The strains overexpressing PA0805.1 and EV were harvested from BM2 glycerol swarm plates with 1% arabinose, and expression was normalized to *rpoD* (*n *=* *5). Download Table S3, DOCX file, 0.02 MB. Copyright © 2020 Coleman et al. 2020 Coleman et al. This content is distributed under the terms of the Creative Commons Attribution 4.0 International license.

### Adherence factors were dysregulated in the PA0805.1 overexpression strain.

**FIG 5:**
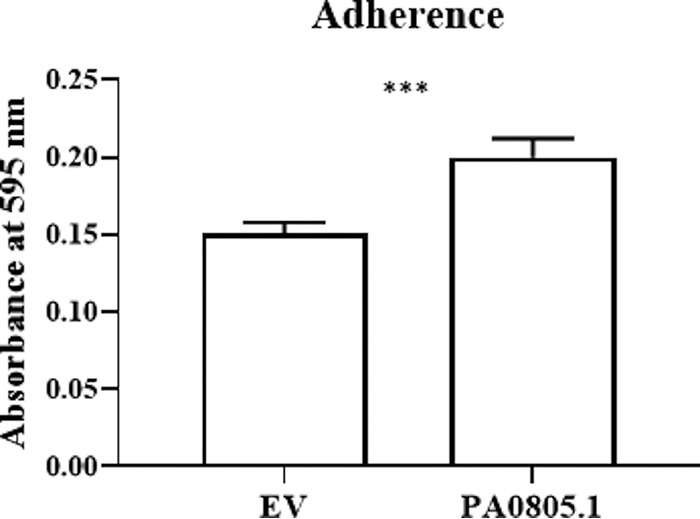
The PA0805.1 overexpression strain demonstrated increased adherence to polystyrene plates in 90% LB with 5% arabinose. Statistically significant differences were determined using Student’s paired *t* test. ***, 0.0001 < *P* ≤ 0.001 (*n *≥* *3).

Among the DE genes were a number of genes that could explain the anti-motility effect. Downregulation of the diguanylate cyclase *fimX* transcript and response regulators *pilGH* could cause the downregulation of twitching motility proteins PilIJK and the type 4 fimbrial biogenesis proteins PilAD, PilMNOPQ, PilEVWX, and PilY1-2 (Table 1). Downregulation of these genes could lead to reductions in twitching and/or swarming motility (Fig. 1 and Table 1) (5). Aside from pilus-related genes, all other adherence factors were upregulated, including the *cupA* gene regulators *cgrABC* but not the *cupA* operon (Table 1). Regulators *rocS1* and *rocR* were also upregulated in the RNA-Seq (Table 1), which can lead to the production of CupB and CupC fimbriae (17). The upregulated genes and proteins also included *cupE1-6*, *cupB2*, *tadABCDGZ*, and *flp* (Table 1). Lastly, the transcriptional regulator *fleR* was also upregulated, along with downstream genes *flgBCDEFGIJ*, *fliCDEF*, and *flhA*. Consistent with this, an adherence assay was performed showing that the overexpression strain PA0805.1 had a modestly increased adherence (Fig. 5). Collectively, the overexpression of these adherence factors and their regulators could influence the reduced motility seen for this strain.

### Additional virulence factors were upregulated in the PA0805.1 overexpression strain.

PA0805.1 also had an increased cytotoxicity against HBE cells (Fig. 2). Among the upregulated DE genes were *lasAB* and *piv*, which are cytotoxic proteases. Other upregulated virulence factors were type VI secretion system (T6SS) genes, *rahU*, alginate and phenazine biosynthetic genes, *pslABCDEFGHIMN*, and *rhlABC* (Table 1 and Table S2). In contrast, certain pyochelin, T1SS, and T3SS genes and proteins were downregulated (Table S2). Several global regulators implicated in virulence could account for these changes, such as LasR and MvaT, but specifically AlgR, ExsD, ExsA, and PcrGH are likely to be involved in regulating alginate and T3SS genes.

### Comparison of RNA-Seq and proteomics.

**FIG 6:**
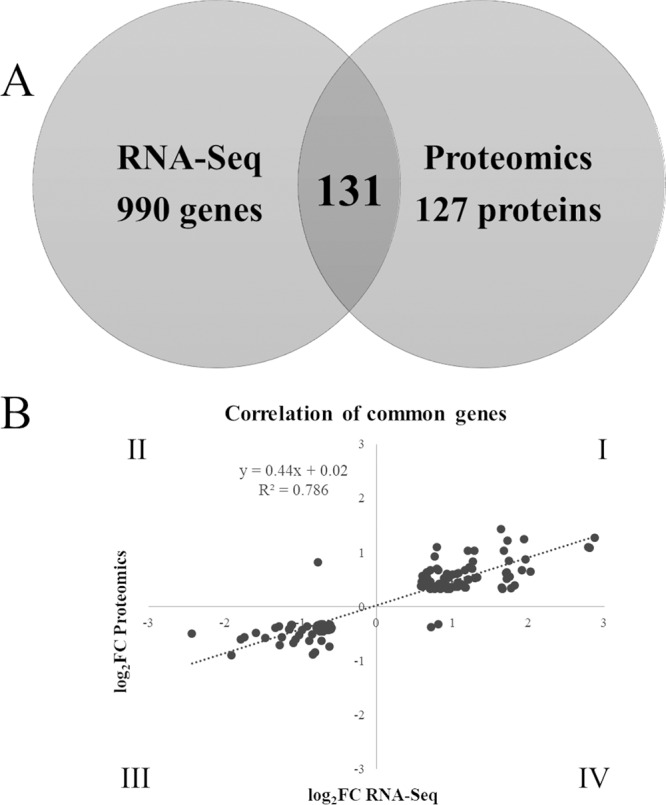
Comparison of RNA-Seq and proteomics data. (A) Venn diagram showing unique and common genes and proteins between the two methods; (B) scatterplot showing log_2_ FC values for the common genes and proteins.

Comparison of the transcriptional and proteomic response revealed considerable overlap, with 131 genes and the encoded proteins identified to be differentially expressed by both methods (Fig. 6A). Of the 131 common gene and protein candidates, there was a good correlation in the direction of fold change (Fig. 6B, *R*^2^ = 0.79), with 128 genes similarly downregulated (quadrant III, 46 genes), or upregulated (quadrant I, 82 genes), while 3 were regulated in opposite fashions (quadrants II and IV). This might relate in part to the differing abilities of the two methods since RNA-Seq was more sensitive and detected in total transcription from 5,194 genes, while proteomics identified in total 2,366 proteins (regardless of differential abundance). It is worth noting that transcripts for extracellular proteins were more likely detected in the RNA-Seq data since wash steps were employed prior to proteomics. Conversely, since sRNAs act by posttranscriptional regulation, it was expected that there would be changes in protein abundance with no corresponding difference in RNA transcript levels, while a single translationally dysregulated regulatory protein might control the expression of hundreds of genes. Lastly, due to the fact that transcripts and proteins have different half-lives, a good correlation between RNA-Seq and proteomic data is not necessarily expected (18, 19). A comparison of RNA-Seq and proteomics using only a cutoff *P* value of ≤0.05 for proteomics is shown in Fig. S3.

10.1128/mSystems.00204-20.3 FIG S3 Comparison of RNA-Seq and proteomics data using a cutoff *P* value of ≤0.05 for proteomics and cutoff *P* and absolute FC values of ≤0.05 and ≥1.5, respectively, for RNA-Seq. (A) Venn diagram showing unique and common genes and proteins between the two methods; (B) scatterplot showing log_2_ FC values for the common genes and proteins. Download FIG S3, TIF file, 0.4 MB. Copyright © 2020 Coleman et al. 2020 Coleman et al. This content is distributed under the terms of the Creative Commons Attribution 4.0 International license.

### In its native state, PA0805.1 contributed to tobramycin susceptibility under swarming conditions.

**FIG 7:**
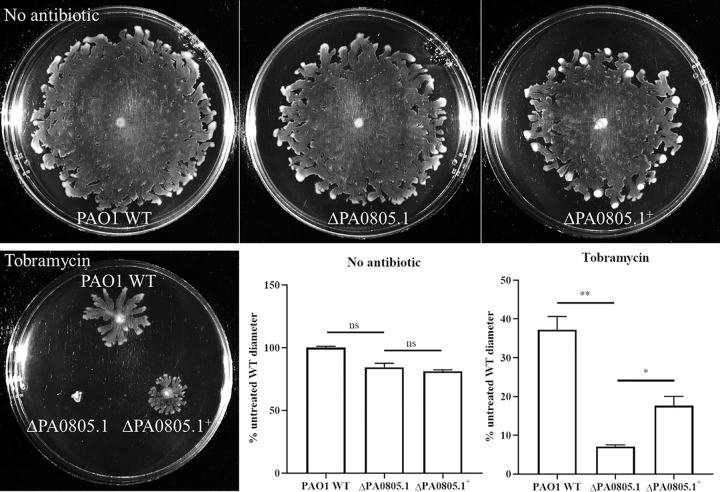
A deletion mutant of PA0805.1 was more susceptible to tobramycin than the parent WT, as assessed in BM2 0.4% glucose swarming agar with no arabinose. The deletion mutant was complemented with a chromosomal insertion of the sRNA PA0805.1. Tobramycin was incorporated into the agar where indicated at 1 μg/ml, and plates were incubated an additional 7 to 8 h (in total, ∼24 h) to observe WT swarming in the presence of tobramycin. Statistically significant differences were determined using paired Student’s *t* test. *, 0.01 < *P* ≤ 0.05; **, 0.001 < *P* ≤ 0.01 (*n *=* *3).

As mentioned above, PA0805.1 was upregulated 5.0-fold ± 1.7-fold under swarming versus swimming conditions (BM2 glucose, normalized to the housekeeping gene 16S rRNA). A deletion mutant of PA0805.1 was constructed and showed no dramatic motility phenotype, but it was more susceptible to tobramycin than the WT under swarming conditions and when complemented substantially restored tobramycin resistance (Fig. 7). These data were consistent with the positive regulation of tobramycin resistance but negative regulation of motility.

## DISCUSSION

Here we demonstrate that the overexpression of sRNA PA0805.1 led to a wide range of phenotypic changes, including reduced swarming, swimming, and twitching motility, as well as increased adherence, cytotoxicity, and tobramycin resistance. In contrast to this situation, few phenotypes were observed for the deletion mutant ΔPA0805.1. This was likely due to the fact that sRNAs usually act by inhibiting translation of target mRNA; hence, overexpression is more likely to have an effect than deletion. As expected, the tobramycin phenotype of the deletion mutant (TOB supersusceptible, Fig. 7) was the opposite of that of the overexpression strain (TOB resistant) under conditions of low expression (using glucose to inhibit expression from the P_BAD_ promoter of pHERD20T) (Fig. 3). The tobramycin phenotype was difficult to observe in the overexpression strain at higher levels of expression (i.e., with arabinose) due to the inhibition of swarming motility.

The differential abundance of many global transcriptional regulators was intriguing and suggests a prospective key global regulatory role for the sRNA PA0805.1 in influencing other regulators (Fig. 4). ClpD, a ClpA/B protease, was upregulated 1.7-fold in RNA-Seq and 1.9-fold in the proteomics data set (Table 1). Proteases can play an important role in regulation by degrading specific proteins (20), and ClpA can function in a regulatory capacity by degrading protein aggregates (21). In addition, proteases such as Lon protease play a role in protein turnover and the degradation of abnormal proteins and can also have DNA binding activity (20). ClpD was identified as a chaperone for the cleavage of BdlA, a chemotactic transducer (22). BdlA was activated by proteolytic cleavage and was required for biofilm dispersal, indicating an important role in motile-sessile switching (22). The transcript for *bdlA* was also upregulated 1.9-fold (Table 1). ClpD was also required for virulence in a chronic lung infection model in rats (23). ClpD appears to be responsive to oxygen levels, as it was upregulated under microaerobic conditions (24) and was also upregulated under aerobic conditions compared with aerobic conditions with nitrate (25).

Two regulators of type IV pili, PilGH, were downregulated more than 2-fold in the RNA-Seq and 1.5- to 1.6-fold in the proteomics data set (Table 1). This could likely account for the downregulation of many pilus genes observed in Table 1. PilGH are two CheY-like response regulators required for type IV pilus function, where PilG regulates pilus extension and PilH regulates retraction (26, 27).

ExsD, a negative regulator of T3SS, was downregulated 3.8-fold in the RNA-Seq and 1.9-fold in the proteomics data set (Table 1). ExsD binds to ExsA, a transcriptional activator of T3SS, and acts as an antiactivator (the ExsD-ExsA complex lacks DNA binding activity) (28, 29). Regulation of T3SS is complex, and we also observed a strong downregulation of ExsA and downstream T3SS components and effectors (Table 1 and Table S2). This may be a result of *exsA* being even more strongly downregulated (4.8-fold) than *exsD*, and downregulation of *exsD* could potentially be a mechanism to compensate for downregulation of T3SS.

CapB, a cold acclimation protein with predicted DNA binding activity (30), was downregulated 1.5-fold in the RNA-Seq and 1.7-fold in the proteomics data set (Table 1). Beyond a role in adaptation to colder temperatures (31, 32), and the presence of an Anr box upstream of *capB* (33), little is known about this gene.

Other interesting regulators with altered abundance when the sRNA PA0805.1 was overexpressed included the following: AlgR, a global regulator in P. aeruginosa that regulates swarming and twitching motility, virulence, and quorum sensing (34–37); LasR, another global regulator that controls quorum sensing, virulence factor production, and motility (38, 39); MvaT, a global regulator of virulence that influences swarming motility and quorum sensing (40); RhlR, which regulates quorum sensing, virulence, swarming, and biofilm formation (39, 41, 42), and PsrA, a regulator of swarming, biofilm formation, T3SS, and antimicrobial peptide resistance (43–46). The expression of several of these regulators was verified by qRT-PCR (Table S3).

Three *in silico* sRNA target prediction tools, IntaRNA2, RNAPredator, and TargetRNA2, were used to predict sRNA targets for PA0805.1 based on hybridization near the 5′ end of mRNA (Table S4). Unfortunately, the results were not compelling, as they did little to explain the many transcriptomic, proteomic, and phenotypic differences observed for the PA0805.1 overexpression strain, and this highlights how predictive programs can underestimate targets. These differences may more likely be explained by the cumulative effects on many different targets.

10.1128/mSystems.00204-20.7 TABLE S4 sRNA targets predicted *in silico*. Where applicable, FC, *P* values (padj/p) and predictive methods are indicated. Download Table S4, DOCX file, 0.02 MB. Copyright © 2020 Coleman et al. 2020 Coleman et al. This content is distributed under the terms of the Creative Commons Attribution 4.0 International license.

In this study, it was shown that overexpressing the sRNA PA0805.1 resulted in broad transcriptional and proteomic changes, most likely through a hierarchical regulatory cascade (Fig. 4). A total of 106 transcriptional regulators, two-component systems, and sigma and anti-sigma factors were dysregulated at the transcriptomic and/or proteomic level, likely explaining the extensive downstream effects. For example, type IV pili, including regulators PilGH and their equivalent chemosensory system (ChpABCE), were downregulated, which would lead to decreased swarming and twitching motility, although certain other adherence factors were upregulated. The protease ClpD and transducer BdlA may also be involved in this motile-sessile switch. Conversely, many flagellar genes were mildly upregulated, but the decreased production of particular regulators, such as LasR, might explain decreased swimming and swarming. Furthermore, there is a connection between swimming and twitching, since the two-component PilRS system controls flagellar genes and swimming motility (47). Since PilS had altered protein abundance (Table 1), PilS may have affected swimming motility. In addition, many virulence factors, including the genes encoding the cytotoxic proteases *lasAB* and *piv*, were upregulated, likely resulting in the observed increased cytotoxicity. Lastly, the multidrug efflux systems *mexXY* and *mexGHI*-*opmD* were upregulated, which might contribute to tobramycin resistance. The sRNA PA0805.1 thus modulates important adaptations in P. aeruginosa, including motility, virulence, and antibiotic resistance.

## MATERIALS AND METHODS

### Bacterial strains and growth conditions.

P. aeruginosa strain PAO1 H103 was grown in Luria-Bertani broth and BM2 minimal medium (62 mM potassium phosphate buffer [pH 7], 0.5 mM MgSO_4_, 10 μM FeSO_4_, and carbon and nitrogen sources as indicated). LB overnight cultures were diluted 1/50 and grown to mid-log phase (optical density at 600 nm [OD_600_] of 0.3 to 0.6).

### Construction of the overexpression plasmid PA0805.1.

PAO1 WT genomic DNA was isolated as specified in the Qiagen DNeasy blood and tissue kit protocol. Three hundred nanograms of DNA was PCR amplified using the cloning primers PA0805.1 F and PA0805.1 R described in Table S5. The PCR product was gel extracted with the GeneJet gel extraction kit (Thermo Fisher) and TOPO cloned (Invitrogen). The TOPO reaction was transformed into Escherichia coli TOP10 and selected with 50 μg/ml of kanamycin (TOPO). Plasmid was subsequently isolated according to instructions with the Thermo Fisher kit and digested with the restriction endonucleases EcoRI and KpnI. After the fragment was gel extracted, it was ligated into the similarly digested vector pHERD20T with T4 DNA ligase (Thermo Scientific), transformed into TOP10 E. coli, and selected with 100 μg/ml of ampicillin. Plasmid sequences were confirmed by Sanger sequencing at the UBC Sequencing and Bioinformatics Consortium.

10.1128/mSystems.00204-20.8 TABLE S5 Primers used in this study. Download Table S5, DOCX file, 0.02 MB. Copyright © 2020 Coleman et al. 2020 Coleman et al. This content is distributed under the terms of the Creative Commons Attribution 4.0 International license.

### Transformation.

Electrocompetent WT P. aeruginosa PAO1 was transformed with both EV pHERD20T and vector overexpressing PA0805.1 according to the method of Choi et al. (48). The resulting strains are referred to as EV and overexpression strains; where EV was used as a “wild-type” control. Transformants were selected with 300 μg/ml of carbenicillin. Expression from the vector was induced by adding arabinose at the desired concentrations.

### Deletion and complementation of PA0805.1.

A deletion mutant of PA0805.l was constructed using previously described methods, with minor modifications (49). Briefly, PAO1 wild-type genomic DNA was PCR amplified using the primers PA0805.1 A1 and A2 and PA0805.1 B1 and B2 (described in Table S5). After gel extraction of the fragments, a fusion PCR was performed using primers PA0805.1 A1 and B2. The PCR product was then TOPO cloned as described above, then digested with BamHI and XbaI and cloned into the vector pEX18Gm, transformed into the E. coli donor strain ST18, and conjugated into WT PAO1 using LB agar plates with 50 μg/ml of 5-aminolevulinic acid. After, conjugants were selected with 30 μg/ml of gentamicin and then counterselected three times on LB plates with 5% sucrose. The deletion mutant was confirmed by lack of growth on gentamicin plates and PCR of the deleted region.

The PA0805.1 deletion mutant was complemented by cloning PA0805.1 into the vector pUC18miniTn7Tp (using the enzymes KpnI and EcoRI) and then conjugating this construct along with the helper plasmid pTNS3, using the E. coli donor strain ST18, into ΔPA0805.1. After the conjugation, colonies with a chromosomal insertion of PA0805.1 were selected by plating on 250 μg/ml of trimethoprim and confirmed by PCR.

### Motility assays.

The concentration of agar and nitrogen source in BM2 were varied to allow for different kinds of motility. Glucose (0.4% [wt/vol]) was often replaced with an alternative carbon source (as indicated) since glucose represses expression from the P_BAD_ promoter of the plasmid pHERD20T (50). Swimming motility was assayed at 0.25% (wt/vol) agar with 7 mM (NH_4_)_2_SO_4_ as the nitrogen source and 20 mM potassium succinate (pH 7.0) as the carbon source, unless otherwise indicated. For swarming assays, plates were solidified with 0.5% (wt/vol) agar, 0.1% Casamino Acids was used as the nitrogen source, and 0.4% (wt/vol) glycerol was used as the carbon source, unless otherwise indicated. Swimming and swarming BM2 plates were composed of 25 ml of medium per plate and dried for 1 h. In contrast, LB medium was used for twitching motility, with 1% agar and 10 ml of medium per plate, and dried overnight. Arabinose was included where indicated for plasmid induction. All plates were stab (swim and twitch) or spot (swarm) inoculated with 1.5 μl of mid-log-phase bacteria. After inoculation, plates were incubated 16 to 20 h at 37°C (unless otherwise indicated) and imaged on the ChemiDoc touch imaging system (Bio-Rad).

### Harvesting bacteria for RNA-Seq and proteomics.

Swarming BM2 plates containing 0.4% (wt/vol) glycerol, 1% (wt/vol) arabinose, and 0.1% (wt/vol) Casamino Acids were grown for 20 h at 37°C. For RNA isolation, the edge of the swarm front was harvested with a plastic loop and transferred to RNAprotect bacteria reagent (Qiagen), pelleted, and stored at −80°C. For protein isolation, the edge of the swarm front was harvested with a plastic loop and transferred to phosphate-buffered saline (PBS; pH 7.4), washed three times with PBS, and stored as a pellet at −80°C.

### RNA isolation.

Pellets were lysed by resuspension in 3 mg/ml of lysozyme dissolved in Tris-EDTA (TE) buffer (pH 8.0; Thermo Fisher). RNA isolation then proceeded according to the manufacturer’s instructions using the RNeasy mini kit (Qiagen). Eluted RNA was further purified with the TURBO DNA-free kit (Thermo Fisher). Two independent runs of RNA-Seq were performed with a total of 5 biological replicates for each strain.

### RNA-Seq and identification of differentially expressed genes.

RNA samples were depleted of rRNA using the RiboZero bacterial kit (Illumina). Libraries of cDNA were prepared using the KAPA stranded total RNA kit (Kapa Biosystems) and sequenced on an Illumina HiSeq 2500. Fastq reads, determined using FastQC v0.11.7 and MultiQC v1.6.dev0, for swarming of P. aeruginosa PAO1 were mapped to its genomic sequence using STAR v2.6.1a. Read counts for individual genes were obtained using HTSeq-count v0.9.1. Significantly differentially expressed genes (adjusted *P* value ≤ 0.05 and fold change ≥ ±1.5) were identified using DESEQ2 1.20.0 and were then used for further analysis.

### Protein digestion and quantification.

Bacterial cell pellets were resuspended in lysis buffer (100 mM HEPES [pH 8.5], 4% SDS, 1× Halt protease inhibitor cocktail; Thermo Fisher Scientific). The cells were sonicated three times for 15 s per cycle with 1 min of cooling on ice between each cycle. The insoluble cellular debris was removed by centrifugation at 17,000 × *g* for 10 min. Protein concentration was determined using the Pierce detergent-compatible Bradford assay kit (Thermo Fisher Scientific). All protein samples were processed and handled using the single-pot solid-phase-enhanced sample preparation (SP3) protocol described below. Prior to SP3 treatment, two types of carboxylate-modified SeraMag Speed beads (GE Life Sciences) were combined in a ratio of 1:1 (vol/vol), rinsed, and reconstituted in water at a concentration of 20 μg of solids per μl. Initially, 200 μg of lysate was reduced with 10 mM (final concentration) dithiothreitol for 30 min at 60°C followed by alkylation using 50 mM (final concentration) iodoacetamide for 45 min in the dark at room temperature. After that, 20 μl of the prepared bead mix was added to the lysate and samples were adjusted to pH 7 using HEPES buffer. To promote protein binding to the beads, acetonitrile was added to a final concentration of 70% (vol/vol) and samples were incubated at room temperature on a tube rotator for 18 min. Subsequently, beads were immobilized on a magnetic rack for 1 min. The supernatant was discarded and the pellet was rinsed twice with 200 μl of 70% ethanol and once with 200 μl of 100% acetonitrile while on the magnetic rack. Rinsed beads were resuspended in 65 μl of 50 mM HEPES buffer (pH 8) supplemented with trypsin–Lys-C mix (Promega) at an enzyme-to-protein ratio of 1:25 (wt/wt) and incubated for 16 h at 37°C. After overnight digestion, supernatant containing peptides was transferred into a fresh tube and subsequently measured for peptide yield using the Pierce quantitative fluorometric peptide assay (Thermo Fisher Scientific).

### TMT labeling.

Representative samples containing 85 μg of peptides were adjusted to the same concentration using 50 mM HEPES (pH 8) and labeled with 10-plex tandem mass tag (TMT) reagents (Thermo Fisher Scientific). The TMT10 reporter channels were sequentially assigned in increasing reporter mass as TMT0 to TMT9. Four TMT10 channels (TMT0 to TMT3) were assigned to samples from the EV strain and three TMT10 channels (TMT7 to TMT9) to samples from the PA0805.1 strain. This represented four biological replicates for the EV strain and three replicates for the PA0805.1 strain. In short, 0.8 mg of each TMT channel was first dissolved in 41 μl of dimethyl sulfoxide (DMSO) before addition to the corresponding peptide digests. The labeling reaction mixture was incubated at room temperature for 60 min. Following incubation, samples were quenched for 15 min with the addition of 8 μl of 5% hydroxylamine. Finally, labeled samples were mixed at equal volumes and desalted using SOLA HRP SPE cartridge (Thermo Fisher Scientific) prior to liquid chromatography-tandem mass spectrometry (LC-MS/MS).

### Mass spectrometry data acquisition.

Analysis of TMT-labeled peptide digests was carried out on an Orbitrap Q Exactive HF-X instrument (Thermo Fisher Scientific, Bremen, Germany). The peptide mixture was resuspended in 0.1% formic acid prior to injection. The sample was introduced using an Easy-nLC 1000 system (Thermo Fisher Scientific) at 2 μg per injection. Mobile phase A was 0.1% (vol/vol) formic acid, and mobile phase B was 0.1% (vol/vol) formic acid in 80% acetonitrile (LC-MS grade). Gradient separation of peptides was performed on a C_18_ [Luna C18(2), 3-μm particle size; Phenomenex, Torrance, CA)] column packed in-house in Pico-Frit (100 μm by 30 cm) capillaries (New Objective, Woburn, MA). Peptide separation was done using the following gradient: 3 to 5% increase of phase B over 4 min, 5 to 7% over 3 min, 7 to 25% over 197 min, 25 to 60% over 25 min, and 60 to 90% over 1 min, with final elution of 90% phase B for 10 min at a flow rate of 300 nl/min.

Data acquisition on the Orbitrap Q Exactive HF-X instrument was configured for the data-dependent method using the full MS/DD−MS/MS setup in a positive mode. Spray voltage was set to 1.85 kV, funnel radio frequency (RF) level at 40, and heated capillary at 275°C. Survey scans covering the mass range of 350 to 1,500 *m/z* were acquired at a resolution of 120,000 (at *m/z* 200), with a maximum ion injection time of 60 ms and an automatic gain control (AGC) target value of 3E6. For MS2 scan triggering, up to 20 of the most abundant ions were selected for fragmentation at 32% normalized collision energy, with the intensity threshold kept at 5.7E4. Automatic gain control (AGC) target value for fragment spectra was set at 1E5, which were acquired at a resolution of 45,000, with a maximum ion injection time of 88 ms and an isolation width set at 0.7 *m/z*. Dynamic exclusion of previously selected masses was enabled for 30 s, charge state filtering was limited to 2 to 6, peptide match was set to preferred, and isotope exclusion was on.

### Identification and differential analysis of proteins.

A January 2019 reference database of PAE PAO1 (taxon 208964) was downloaded from UniProt (www.uniprot.org). The one-dimensional (1D) LC-MS run was converted into an MGF file using the Proteome Discoverer bundled tool and was searched against the PAO1 database using X!tandem (cyclone 2012.10.01.1). Peptide identification settings were standard for the instrument: single missed cleavage tryptic peptides were permitted, with a parent and fragment mass tolerance of 10 ppm. A fixed posttranslational modification of C + 57.021 was applied, and variable posttranslational modifications, including N-terminal acetylation, deamidation, phosphorylation, and oxidation, were permitted. Peptide assignment into source proteins was managed by X!tandem.

Peptide level TMT10 reporter tag intensities were integrated across a window of ±3 mDa each and corrected for isotopic overlap between channels using the supplied batch-specific correction matrix. Protein level quantitation required at least two unique peptides with expectation values of log(e) ≤ −1.5 each, yielding highly confident protein assignments of at least log(e) ≤ −3. The sum of peptide level TMT10 reporter tag intensities for each protein was converted into a log_2_ scale for simplified differential analysis. Protein expression values across each TMT10 reporter channel were normalized into a common scale (mean = 0; standard deviation [SD] = 1).

Differential analysis between normalized sample populations (PA0805.1 versus EV) was conducted using the Welch *t* test function in Excel between population averages. The *P* scores were not subjected to multiple-testing corrections, and any differences with *P* values of <0.05 were considered candidates for biological exploration. Differences between normalized population means were scaled back into a log_2_ scale by multiplying them by an average system-wide SD of 2.26.

### qRT-PCR.

Swarming or swimming BM2 plates containing 0.1% (wt/vol) Casamino Acids and the desired carbon source were grown overnight at 37°C. RNA was isolated and DNase digested as described above and quantified on a NanoDrop ND-1000 spectrophotometer. RNA was then diluted to 1 ng/μl and 5 μl was used in a total reaction volume of 25 μl. The qScript one-step SYBR green quantitative reverse transcriptase PCR (qRT-PCR; Quantabio) was used, and samples were run on a LightCycler 96 (Roche). Quantification cycle (*C_q_*) values were normalized to the housekeeping gene *rpoD* or 16S, as indicated, using the threshold cycle (ΔΔ*C_T_*) method. qPCR primers used are described in Table S5.

### Tobramycin kill curve.

Tobramycin kill curves were performed as previously described (4), with minor modifications. Briefly, bacteria were harvested from BM2 glucose swarm (0.5% agar) and swim (0.3% agar) plates in 62 mM potassium phosphate buffer (pH 7.0) and treated with 20 μg/ml of tobramycin in a 5-ml volume with aeration at room temperature.

### MIC assay.

Bacteria were seeded at 5 × 10^5^ CFU/ml in a 2-fold concentration gradient of antibiotic in BM2 with 0.4% (wt/vol) glucose and 0.1% (wt/vol) Casamino Acids and no (NH_4_)_2_SO_4_ at 100 μl/well in 96-well polystyrene round-bottom plates. After 24 h of incubation at 37°C, the minimal concentration to inhibit visible bacterial growth was reported as the MIC.

### Adherence assay.

Overnight cultures were diluted to a final OD_600_ of 0.03 in 90% LB supplemented with 5% (wt/vol) arabinose and seeded at 100 μl/well in 96-well flat-bottom polystyrene plates. After 4 h of incubation at 37°C, unattached cells were removed by discarding the media and rinsing three times with distilled water (dH_2_O). A total of 105 μl of 0.1% crystal violet was added and incubated with shaking for 20 min at room temperature, then the plates were rinsed three times with dH_2_O, and the crystal violet was solubilized by adding 110 μl of 70% (vol/vol) ethanol and shaking for 20 min at room temperature. Then the absorbance at 595 nm was read in an Epoch plate reader (BioTek).

### Cytotoxicity against HBE cells.

Human bronchial epithelial 16HBE14o- (HBE) cells between passages 14 and 40 were grown in minimum essential medium with Earle’s salts (1×) (MEM; Gibco) supplemented with 10% fetal bovine serum (FBS; Gibco), 2 mM l-glutamine (Gibco), and 1% penicillin-streptomycin (Gibco). After cells reached 80 to 100% confluency, they were washed once with PBS (pH 7.4, 1×; Gibco), trypsinized with 0.25% trypsin-EDTA (Gibco), and diluted in medium before counting. HBE cells were seeded at 2 × 10^4^/well in a 96-well plate and grown again to confluency (2 to 3 days). A total volume of 200 μl per well was used. Then the medium was changed to Dulbecco’s modified Eagle medium with l-glutamine and no d-glucose (DMEM; Gibco) supplemented with 1% FBS and 1% sodium pyruvate 1 to 2 h prior to infection. Next, bacterial cultures were prepared by pelleting overnight cultures, washing once with PBS, and resuspending in DMEM (no glucose)-1% FBS-1% sodium pyruvate with or without 1% (wt/vol) arabinose. Bacteria were diluted in the same medium. Next, the medium of the HBE cells was removed and replaced with a suspension containing 3 × 10^5^ CFU/ml of bacteria. The coculture was incubated at 37°C with 5% CO_2_ for 16 h, followed by monitoring the release of lactate dehydrogenase (LDH) as an indicator of cytotoxicity as described below. Cells treated with 2% (vol/vol) Triton X-100 (Fisher Scientific) in DMEM (no glucose)-1% FBS-1% sodium pyruvate were used as a positive control for the LDH assay.

### Cytotoxicity assay for LDH activity.

Plates were centrifuged for 5 min at 1,000 rpm in an Eppendorf 5810 R centrifuge (15 A version), and 50 μl of supernatant was removed and mixed with 50 μl of solution as indicated in the cytotoxicity detection kit (Roche) assessing release of LDH (1/100 catalyst/reaction mixture) and incubated for 10 min at room temperature in the dark. Then the absorbances at 492 and 900 nm were read in the Epoch plate reader (BioTek). Next, the absorbance at 900 nm was subtracted from the absorbance at 492 nm. Percent cytotoxicity was calculated by subtracting controls (HBE cells alone, and bacteria alone) from coculture values and then normalizing to the Triton X-100 control.

### Growth curves.

Overnight cultures were diluted to a final OD_600_ of 0.05 in each of the three media listed below and seeded in 96-well round-bottom plates at 100 μl/well. They were incubated at 37°C with shaking at a frequency 567 cpm (3 mm) in a Synergy H1 microplate reader, and the OD_600_ was read every 30 min. Media used were (i) liquid BM2 swarming medium (with 0.4% [wt/vol] glycerol, 0.1% [wt/vol] Casamino Acids, and 1% [wt/vol] arabinose), (ii) 90% LB with 5% arabinose, and (iii) DMEM with no glucose but with 1% FBS, 1% sodium pyruvate, and 1% arabinose.

### *In silico* sRNA target prediction.

sRNA targets were predicted using three tools: IntaRNA2 (51), RNAPredator (52), and TargetRNA2 (53). For IntaRNA2 and TargetRNA2, input parameters were adjusted to 75 nucleotides up- and downstream, and a minimum of 7 bp in the seed sequence was used. Cutoffs used were top 100 and *P ≤ *0.05 for IntaRNA2, *P ≤ *0.05 for TargetRNA2, and top 100 for RNAPredator. Only targets predicted by more than one tool were considered.

### Data availability.

RNA-Seq data were deposited in GEO under the accession number GSE137738. Proteomics data were deposited in MassIVE under index number MSV000084373.
