# Supplementary figures and images for: Overexpression of the Small RNA PA0805.1 in Pseudomonas aeruginosa Modulates the Expression of a Large Set of Genes and Proteins, Resulting in Altered Motility, Cytotoxicity, and Tobramycin Resistance
Source: mSystems. 2020 May 19;5(3):e00204-20. doi: 10.1128/mSystems.00204-20 (PMC7253367; doi:10.1128/mSystems.00204-20)

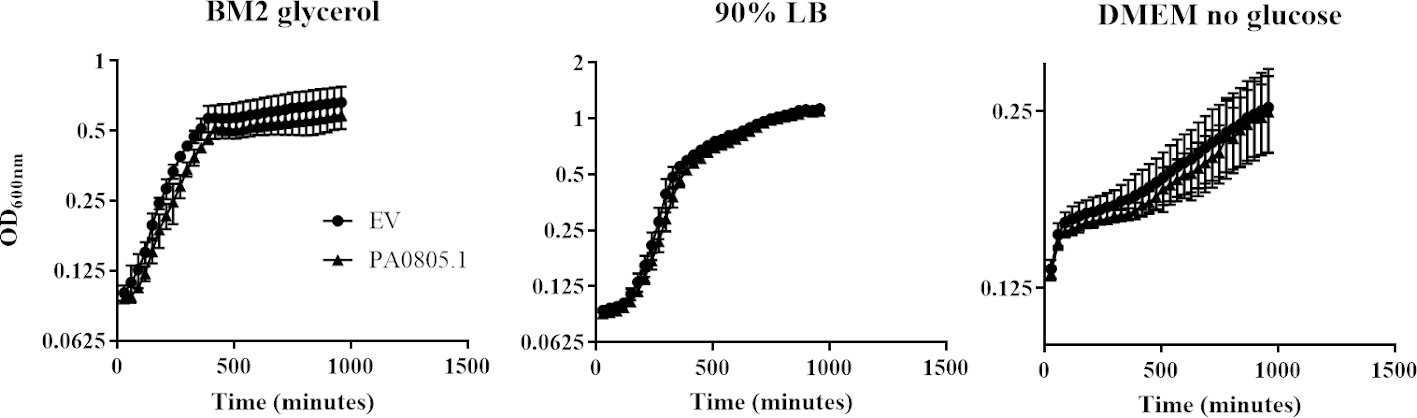

Supplement: FIG S1 [file mSystems.00204-20-sf001.tif]

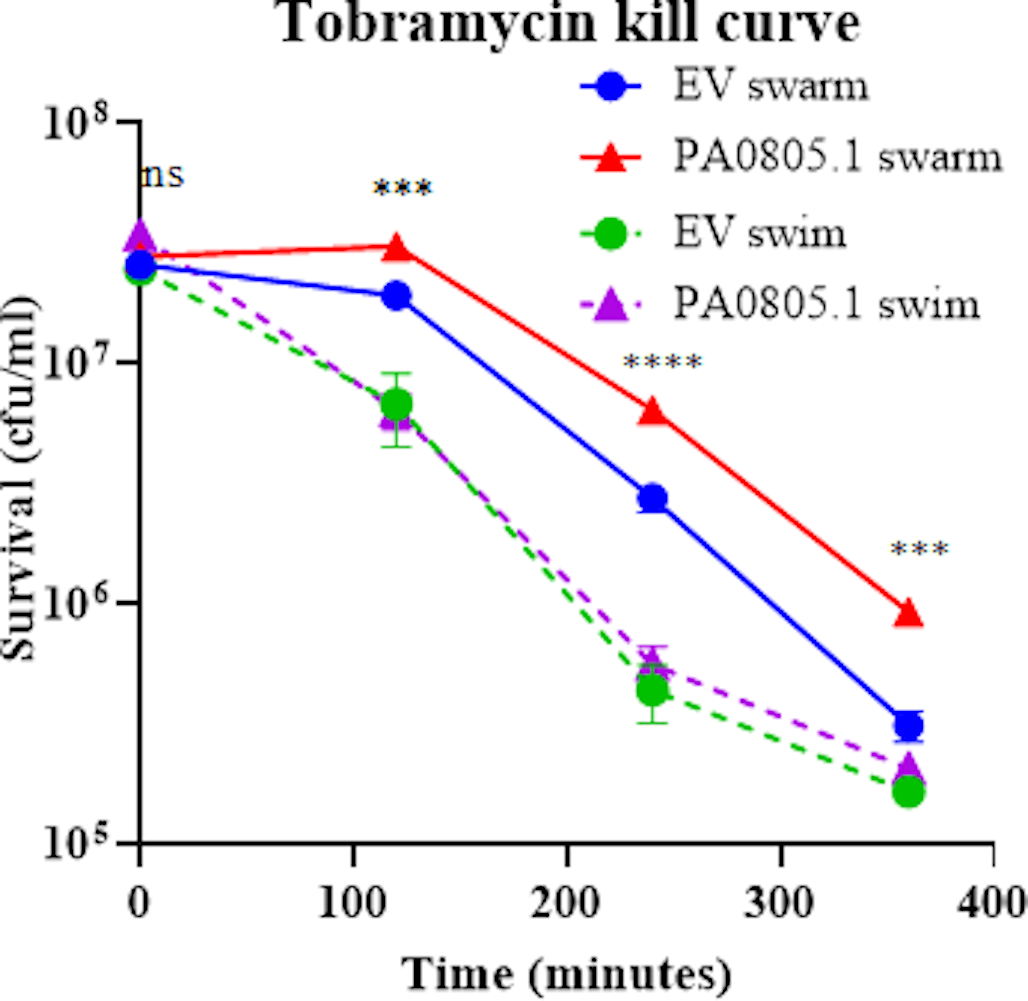

Supplement: FIG S2 [file mSystems.00204-20-sf002.tif]

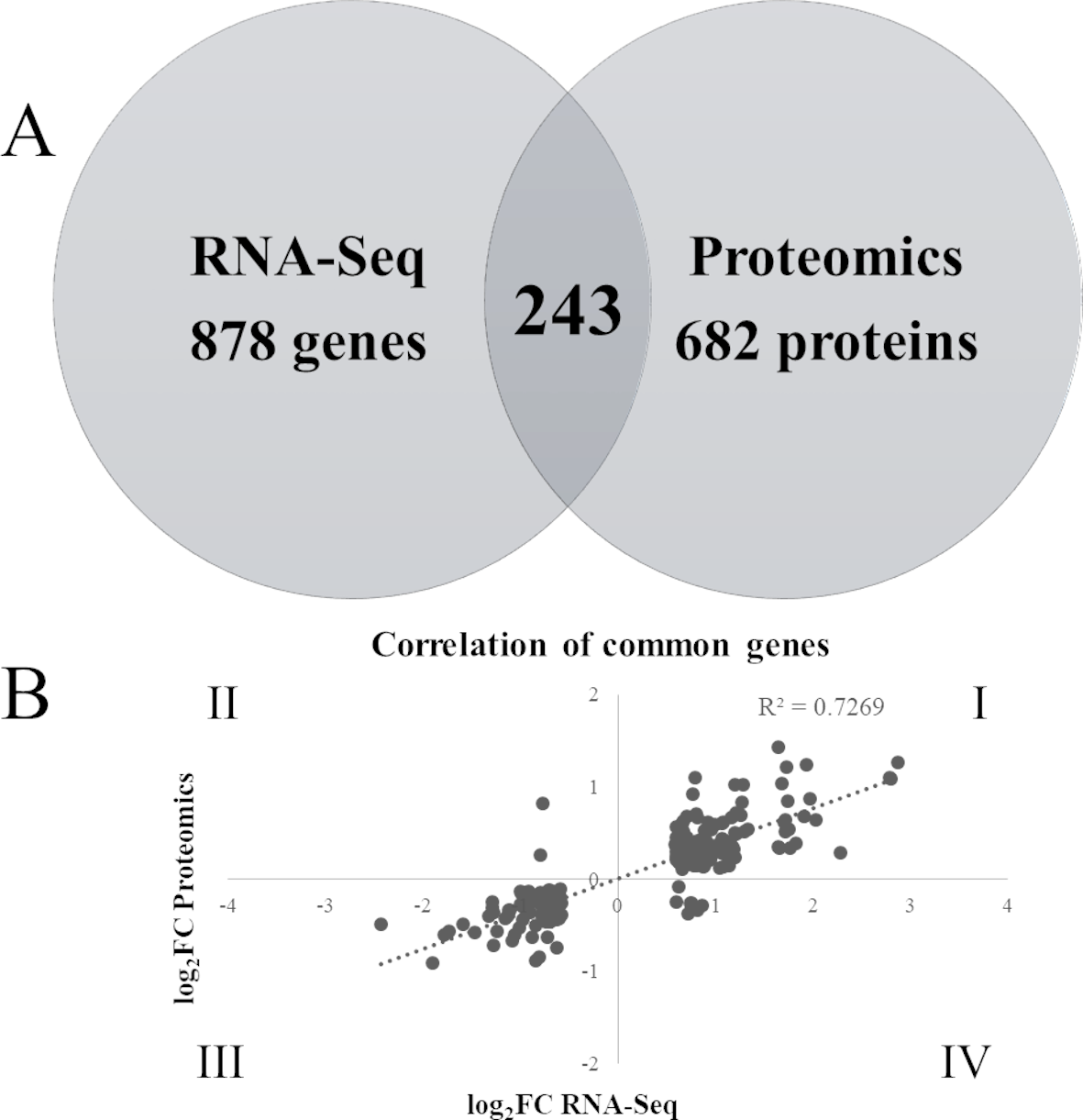

Supplement: FIG S3 [file mSystems.00204-20-sf003.tif]
