# Supplementary material for: Overexpression of the Small RNA PA0805.1 in Pseudomonas aeruginosa Modulates the Expression of a Large Set of Genes and Proteins, Resulting in Altered Motility, Cytotoxicity, and Tobramycin Resistance
Source: mSystems. 2020 May 19;5(3):e00204-20. doi: 10.1128/mSystems.00204-20 (PMC7253367; doi:10.1128/mSystems.00204-20)
Supplement: TABLE S1 [file mSystems.00204-20-st001.docx]

| **Strain** | **Plasmid** | **MIC** |
| --- | --- | --- |
| PAO1 | pHERD20T (EV) | 1-2 |
| PAO1 | PA0805.1 | 2 |
| PAO1 WT | - | 1-2 |
| ΔPA0805.1 | - | 1-2 |
| ΔPA0805.1^+^ | Chromosomal insertion of PA0805.1 | 1-2 |
