# Supplementary material for: Overexpression of the Small RNA PA0805.1 in Pseudomonas aeruginosa Modulates the Expression of a Large Set of Genes and Proteins, Resulting in Altered Motility, Cytotoxicity, and Tobramycin Resistance
Source: mSystems. 2020 May 19;5(3):e00204-20. doi: 10.1128/mSystems.00204-20 (PMC7253367; doi:10.1128/mSystems.00204-20)
Supplement: TABLE S3 [file mSystems.00204-20-st003.docx]

| **Gene** | **FC** |
| --- | --- |
| *algR* | 2.0 ± 0.3 |
| *exsE* | -1.8 ± 0.3 |
| *mexX* | 3.1 ± 0.6 |
| PA0618 | 4.7 ± 1.1 |
| *psrA* | 3.3 ± 1.3 |
| *rhlR* | 2.7 ± 0.4 |
| *soxR* | 1.5 ± 0.1 |
