# Supplementary material for: Overexpression of the Small RNA PA0805.1 in Pseudomonas aeruginosa Modulates the Expression of a Large Set of Genes and Proteins, Resulting in Altered Motility, Cytotoxicity, and Tobramycin Resistance
Source: mSystems. 2020 May 19;5(3):e00204-20. doi: 10.1128/mSystems.00204-20 (PMC7253367; doi:10.1128/mSystems.00204-20)
Supplement: TABLE S4 [file mSystems.00204-20-st004.docx]

| **Locus** | **Name** | **Description** | **RNA-Seq** | | **Proteomics** | | **Predicted by** |
| --- | --- | --- | --- | --- | --- | --- | --- |
|  |  |  | **FC** | **padj** | **FC** | **p** |  |
| PA0142 |  | hypothetical protein |  |  |  |  | TargetRNA2, IntaRNA2 |
| PA0564 |  | probable transcriptional regulator |  |  |  |  | TargetRNA2, IntaRNA2 |
| PA1248 | *aprF* | alkaline protease secretion outer membrane protein AprF precursor |  |  | -1.18 | 4.6E-03 | RNAPredator, IntaRNA2 |
| PA1644 |  | conserved hypothetical protein |  |  |  |  | RNAPredator, IntaRNA2 |
| PA1912 | *femI* | ECF sigma factor |  |  |  |  | TargetRNA2, RNAPredator |
| PA2619 | *infA* | initiation factor |  |  |  |  | RNAPredator, IntaRNA2 |
| PA2677 | *hplR* | probable type II secretion protein |  |  |  |  | RNAPredator, IntaRNA2 |
| PA2783 | *mep72* | metzincin protease | -2.45 | 9.2E-05 |  |  | RNAPredator, IntaRNA2 |
| PA3105 | *xcpQ* | general secretion pathway protein D |  |  |  |  | RNAPredator, IntaRNA2 |
| PA3339 | *plpD* | patatin-like protein |  |  |  |  | TargetRNA2, IntaRNA2 |
| PA3840 | *ybiN* | putative rRNA methyl transferase | -1.87 | 2.6E-07 |  |  | RNAPredator, IntaRNA2 |
| PA4539 |  | hypothetical protein |  |  |  |  | RNAPredator, IntaRNA2 |
| PA5157 | *marR* | probable transcriptional regulator |  |  |  |  | TargetRNA2, RNAPredator |
| PA5187 |  | probable acyl-CoA dehydrogenase |  |  | 1.11 | 3.5E0-3 | TargetRNA2, IntaRNA2 |
