# Supplementary material for: Overexpression of the Small RNA PA0805.1 in Pseudomonas aeruginosa Modulates the Expression of a Large Set of Genes and Proteins, Resulting in Altered Motility, Cytotoxicity, and Tobramycin Resistance
Source: mSystems. 2020 May 19;5(3):e00204-20. doi: 10.1128/mSystems.00204-20 (PMC7253367; doi:10.1128/mSystems.00204-20)
Supplement: TABLE S5 [file mSystems.00204-20-st005.docx]

| **Name** | **Purpose** | **Sequence (5’ -> 3’)** |
| --- | --- | --- |
| PA0805.1 A1 | cloning | ctcggatccTCTGAGTGGAGTACGGGAGA |
| PA0805.1 A2 | cloning | CGAAAGATATACAATCCGGGAAAGCGTGAAAGTAAAGGAACAT |
| PA0805.1 B1 | cloning | ATGTTCCTTTACTTTCACGCTTTCCCGGATTGTATATCTTTCG |
| PA0805.1 B2 | cloning | gactctagaGAAGGATGGGAACAGGTCG |
| PA0805.1 F | cloning | gactctagaATGGAGCAGCGTATATTGC |
| PA0805.1 R | cloning | ctcggtaccCTGCGTACCAAACTGAAAGTC |
| 16S F | qPCR | ggcaggcctaacacatgcaa |
| 16S R | qPCR | ttatcccccactaccaggca |
| *algR* F | qPCR | CGATATCGTCCTGCTGGATA |
| *algR* R | qPCR | GCCGTGCAGAAGATCACA |
| *exsE* F | qPCR | AATCGATTTCGCCGGTGCAG |
| *exsE* R | qPCR | ACGGTCCTCGCCGCGAACGG |
| *mexG* F | qPCR | ACTCGCTCGAAAGCAACTGG |
| *mexG* R | qPCR | AGGCTGGCCTGATAGTCGAA |
| *mexH* F | qPCR | ATCCGTCTCAAGGCGCAGTT |
| *mexH* R | qPCR | TTGTCCAGCTGTTCCTGCGA |
| *mexI* F | qPCR | ATCACCGTCACCACCGAGTA |
| *mexI* R | qPCR | AAAGGTAGTCGATGCCCTCC |
| *mexX* F | qPCR | GTGCAGAACCGCCTGAAGAT |
| *mexX* R | qPCR | GTGACGATCAGCTGGATGCT |
| *opmD* F | qPCR | TACAGCCGCAGCATCGAACA |
| *opmD* R | qPCR | CCGAACAGGTCGATTTCCCA |
| PA0618 F | qPCR | AAGAGCTGTTGGGCGACTTC |
| PA0618 R | qPCR | GCCAGTTGCAGAAGCTTGAG |
| PA0805.1 qF | qPCR | TGGTATTGCGGGACGCC |
| PA0805.1 qR | qPCR | ACTCTTCTGAAGCAATCCCCTG |
| *psrA* F | qPCR | GGAAAAGGAGCTGGATCGTC |
| *psrA* R | qPCR | AGCGCATGAAGATCGACAG |
| *rhlR* F | qPCR | CGCGTCGAACTTCTTCTGGAT |
| *rhlR* R | qPCR | GCAAGAGTTCCGGGGAAATC |
| *rpoD* F | qPCR | TCACGCACGCAGAGTTGCAT |
| *rpoD* R | qPCR | AAGCTGGTGCCCAAGCAGTT |
| *soxR* F | qPCR | GAACTGAGCGTCGGCGAACTGG |
| *soxR* R | qPCR | GTTGGTCGCGCAACAGCAGCAG |
